# Molecular Mechanism Underlying Derepressed Male Production in Hexaploid Persimmon

**DOI:** 10.3389/fpls.2020.567249

**Published:** 2020-12-22

**Authors:** Kanae Masuda, Naoko Fujita, Ho-Wen Yang, Koichiro Ushijima, Yasutaka Kubo, Ryutaro Tao, Takashi Akagi

**Affiliations:** ^1^Graduate School of Environmental and Life Science, Okayama University, Okayama, Japan; ^2^Department of Crop Sciences, University of Illinois at Urbana-Champaign, Urbana, IL, United States; ^3^Graduate School of Agriculture, Kyoto University, Sakyo-ku, Japan

**Keywords:** monoecious, sex expression, polyploidy, Oriental persimmon, co-expression network

## Abstract

Sex expression in plants is often flexible and contributes to the maintenance of genetic diversity within a species. In diploid persimmons (the genus *Diospyros*), the sexuality is controlled by the Y chromosome-encoded small-RNA gene, *OGI*, and its autosomal counterpart, *MeGI*. Hexaploid Oriental persimmon (*Diospyros kaki*) evolved more flexible sex expression, where genetically male individuals carrying *OGI* can produce both male and female flowers (monoecy). This is due to (semi-)inactivation of *OGI* by the *Kali*-SINE retrotransposon insertion on the promoter region and the resultant DNA methylations. Instead, flower sex determination in Oriental persimmon is also dependent on DNA methylation states of *MeGI*. Here, we focused on a cultivar, Kumemaru, which shows stable male flower production. Our results demonstrated that cv. Kumemaru carries *OGI* with *Kali*-SINE, which was highly methylated as well as in other monoecious cultivars; nevertheless, *OGI* gene could have a basal expression level. Transcriptomic analysis between cv. Kumemaru and 14 cultivars that predominantly produce female flowers showed differentially expressed genes (DEGs) specific to cv. Kumemaru, which is mainly involved in stress responses. Co-expression gene networks focusing on the DEGs also suggested the involvement of stress signals, mainly via gibberellin (GA), salicylic acid (SA), and especially jasmonic acid (JA) signal pathways. We also identified potential regulators of this co-expression module, represented by the TCP4 transcription factor. Furthermore, we attempted to identify cv. Kumemaru-specific transcript polymorphisms potentially contributing to derepressed *OGI* expression by cataloging subsequences (k-mers) in the transcriptomic reads from cv. Kumemaru and the other 14 female cultivars. Overall, although the direct genetic factor to activate *OGI* remains to be solved, our results implied the involvement of stress signals in the release of silenced *OGI* and the resultant continuous male production.

## Introduction

Sexuality is a main biological process that maintains genetic diversity within a species. Nevertheless, most angiosperms are hermaphroditic, which is thought to be the ancestral state in flowering plants (Ainsworth, 2000). Minorities have evolved distinct sexual systems, including monoecy (i.e., the formation of male and female flowers by one plant) or dioecy (i.e., separate male and female plants) (Renner, 2014). Regarding dioecy, a few genetic determinants have been identified in some lineages, including persimmons (*Diospyros* spp.) (Akagi et al., 2014), garden asparagus (*Asparagus officinalis* L.) (Murase et al., 2017; Harkess et al., 2017; Tsugama et al., 2017), and kiwifruit (*Actinidia* spp.) (Akagi et al., 2018, 2019). Candidates of sex determinants have been also identified in date palm (Torres et al., 2018), wild strawberries (Tennessen et al., 2018), grape (Zhou et al., 2017), and poplar (Müller et al., 2020). These studies have revealed molecular bases of the genetic sex-determining genes and the evolutionary paths to establish dioecious sex determination systems. On the other hand, mechanisms relating to transitions out of dioecy have been little understood. Transition out of dioecy often involves domestication or polyploidizations in plants (Comai, 2005; Goldberg et al., 2017; Henry et al., 2018). The transition from dioecy into hermaphroditism in domestication would be explainable with artificial selective pressures for stable production, such as in papaya and grape (VanBuren et al., 2015; Zhou et al., 2019). Yet, a distinct mechanism to connect polyploidization and transition out of dioecy remains to be solved.

**FIGURE 1:**
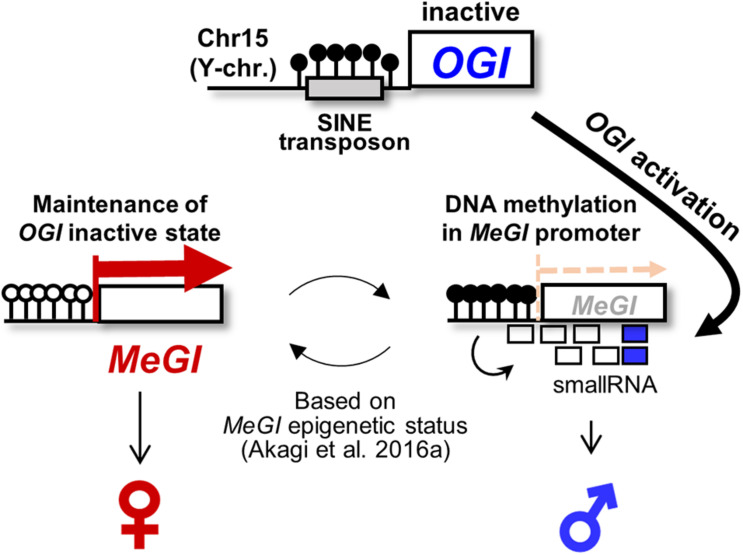
Epigenetic sex determination model in monoecious *D. kaki*. The Y chromosome-encoded small-RNA gene *OGI* triggers to accumulate the small-RNAs targeting its autosomal counterpart *MeGI*, which induces DNA methylation of the *MeGI* sequence, resulting in the repression of the *MeGI* expression. In *D. kaki* (Oriental persimmon), *OGI* is basically inactivated by the *Kali*-SINE insertion and the resultant DNA methylation. Hypothetically, occasional (or accidental) activation of *OGI* allows small-RNA production and DNA methylation in *MeGI*. Once the promoter region of *MeGI* is methylated, the fate of flower sexuality depends on the maintenance or release of DNA methylation in *MeGI* (Akagi et al., 2016a).

In dioecious *Diospyros* species, the Y chromosome-encoded small-RNA (smRNA) gene, *OGI*, which is believed to be a single-sex determinant, is responsible for repressing the expression of the autosomal counterpart, *MeGI* (Akagi et al., 2014; Yang et al., 2019). A recent polyploidization event in wild diploid species that generated the cultivated hexaploid Oriental persimmon (*Diospyros kaki*) promoted a transition out of dioecy and established a plastic sex-determination system (Akagi et al., 2016a; Henry et al., 2018). In hexaploid persimmon, genetically male plants carrying at least one copy of the *OGI* gene exhibit monoecy, while individuals carrying only X chromosomes are female (Akagi et al., 2016a,b). The basic mechanism for producing the male flower in hexaploid monoecious *D. kaki* individuals is identical to that in diploid dioecious male individuals, in which it depends on the repression of *MeGI* expression. In hexaploid *D. kaki*, *OGI* expression is, however, substantially inactivated because of the presence of a highly methylated SINE-like transposon, named *Kali*, in the promoter region (Akagi et al., 2016a). Hypothetically, once *OGI* occasionally (or accidentally) works to trigger small-RNA production in *MeGI*, the resultant DNA methylation in the promoter region represses *MeGI* expression to form initial male flowers. Then, from the male parental branches, the sexuality of the derived flowers is dependent on the maintenance or release of DNA methylation in *MeGI*. These situations derive monoecious individuals that are genetically male (Figure 1; Akagi et al., 2016a; Henry et al., 2018). In previous studies, all of the assessed genetically male cultivars/accessions, which carry at least one copy of *OGI* gene, included the *Kali* insertion in the *OGI* promoter region and exhibited monoecy or female (Akagi et al., 2016a,b). This suggested that the derepression of *OGI* had importance in the evolutionary path to establishing the hexaploid *D. kaki* population.

Among a wide variety of Oriental persimmon cultivars deposited to the germplasm repository in the Genebank Project, NARO, Japan,^1^ cv. Kumemaru^2^ is thought to specifically produce only male flowers. This cultivar was derived from a selection of chance seedling in the purpose of the collection of stable pollinizers at Toyama prefecture in Japan. Although the genetic factors or inheritance mode involving this derepressed male production system have not been identified, the previous study has already indicated that cv. Kumemaru carries the *OGI* gene with *Kali*-SINE insertion in its promoter region, as well as in other cultivars (Akagi et al., 2016a). To characterize the mechanism underlying the derepressed male production in cv. Kumemaru, in this study, we assessed on *OGI* regulation and then approached from co-expression network and comprehensive polymorphism detection. Insights into an irregularly derepressed male production system would shed light on the mechanism of the repressing in hexaploid persimmon.

## Materials and Methods

### Plant Materials

Developmental stages of diploid (*Diospyros lotus*) and Oriental persimmon (*D. kaki* Thunb.) buds/flowers were defined in previous studies (Akagi et al., 2016a). Flower buds in 14 predominantly female-producing cultivars, which carry at least one copy of *OGI* but have produced no or rare male flowers, and cv. Kumemaru were collected in early June 2017, which correspond to the male/female primordia differentiation (Akagi et al., 2014, 2016a). The 13 dominantly female-producing cultivars (cv. Atago, cv. Beniwase, cv. Chagone, cv. Kunitomi, cv. Nagara, cv. Nanshi, cv. Ogosho, cv. Okugosho, cv. Oniwa, cv. Saisho, cv. Suruga, cv. Yashima, cv. Yoshino) planted in the experimental orchard of Kyoto University (Kyoto, Japan) and cv. Kumemaru and cv. Suruga planted in the grape and persimmon research station, NIFTS, NARO, Japan (Hiroshima, Japan), were used. To construct mRNA-Seq libraries, the buds were separately collected at different positions of medial and top in branches with three to four biological replicates abbreviated as #1 and #2, respectively. The collected samples were frozen at −80°C until used for RNA extractions.

### Characterization of *MeGI* and *OGI*

cDNAs of developing flower buds of cvs. Taishu and Kumemaru, and *D. lotus* cv. Kunsenshi-male were synthesized from total RNA with ReverTra Ace qRT-PCR Master Mix (TOYOBO, Osaka, Japan). The relative expression level of *OGI* and *MeGI* (Akagi et al., 2014, 2016b for the primer sequences) was detected by qRT-PCR using a LightCycler 480 (Roche Diagnostics, Mannheim, Germany), with four biological replicates. A constitutively expressed *DkActin* gene in hexaploid persimmon (Akagi et al., 2009) was used for standardizing expression levels. qPCR analyses were conducted under the following conditions: 95°C for 5 min followed by 45 cycles of 95°C for 15 s, 60°C for 15 s, and 72°C for 30 s, using THUNDERBIRD SYBR qPCR Mix (TOYOBO).

Genomic DNA was extracted from young flower buds with the CTAB method (Akagi et al., 2014). The full-length and 5′ promoter regions of the *OGI* in cv. Kumemaru and a wide variety of *Diospyros* species, *D. kaki*, *D. lotus*, *Diospyros glaucifolia*, *Diospyros digyna*, *Diospyros virginiana*, *Diospyros mespiliformis*, and *Diospyros rhombifolia*, were amplified from gDNAs, with the OGI-prom-2F-TOPO and OGI-spR primer set (Akagi et al., 2016a). Quantitative genotyping of *OGI* in cv. Kumemaru was conducted according to the previous report (Akagi et al., 2016b). Alleles of *Kali*-SINE insertion on the *OGI* promoter and *OGI* transcript region, in cv. Kumemaru and *D. kaki* cultivars, were amplified from gDNAs, with primer sets OGI-441-kali-F (5′- GCTCCAGTTTAGTTATGGAAGAG-3′, T_*m*_: 60) and OGI-441-kali-R (5′- ATTGCCAACGTTCACATCAC -3′, T_*m*_: 63) for the Kali-SINE, and OGI-candF and OGI-spR for the *OGI* transcript region (Akagi et al., 2016b). For the detection of DNA methylation status in *OGI*, gDNA extracted from developing flower buds was treated with an EZ DNA Methylation-Gold kit (Zymo Research, United States) to deaminate and convert non-methylated cytosine residues into uracil residues. Bisulfite-treated *OGI* 5′ promoter sequences were amplified with Epi Taq HS (TaKaRa) and each four sense- and antisense-direction primer sets (Akagi et al., 2016a). The bisulfite amplicons were subjected to Illumina library construction described below.

#### Illumina Sequencing

To construct mRNA-Seq libraries, total RNA was purified with the Dynabeads mRNA Purification Kit (Invitrogen, United States). The mRNA-Seq libraries were prepared with the KAPA RNA HyperPrep Kit (Roche, Switzerland) as previously described (Yang et al., 2019), followed by a DNA cleanup step with AMPure XP beads (Beckman Coulter, United States; AMPure: reaction = 0.8:1). To construct bisulfite-PCR-Seq libraries, the promoter region of *OGI* was amplified from bisulfite-treated gDNA of cv. Kumemaru, according to the previous report (Akagi et al., 2016a). The bisulfite amplicons were purified with AMPure and then applied to KAPA HyperPrep Kit (Roche) as previously described (Akagi et al., 2016a). The purified libraries were quantified with a Qubit 2.0 fluorometer (Invitrogen) and then analyzed with the Illumina HiSeq 4000 system at the QB3 Genomic Sequencing Laboratory of UC Berkeley.^3^ The resulting SR50 sequencing reads were analyzed at the Vincent J. Coates Genomics Sequencing Laboratory at UC Berkeley. Raw sequencing reads were processed using custom Python scripts developed in the Comai Laboratory, which are available online,^4^ as previously described (Akagi et al., 2014). All Illumina sequencing data have been deposited in the DDBJ database: Short Read Archives (SRA) database (BioProject ID PRJDB9564, Run ID DRR233540-DRR233569).

### Transcriptomic Data Profiling

The mRNA-Seq Illumina reads were aligned to the reference coding sequences (CDSs) of the diploid Caucasian persimmon, *D. lotus*^5^ (Akagi et al., 2020), using the default parameters of the Burrows-Wheeler Aligner (BWA) (version 0.7.12) (Li and Durbin, 2009).^6^ These settings allow the mapping of variable allele sequences in hexaploid *D. kaki* (Yang et al., 2019). The read counts per CDS were determined from the aligned SAM files using a custom R script to calculate the RPKM for each gene. To examine the gene expression dynamics in 14 predominantly female-producing cultivars and cv. Kumemaru, a PCA was conducted using the genes with RPKM > 1, with prcomp in R.

Differentially expressed genes (DEGs) between predominantly female-producing cultivars and cv. Kumemaru were detected by DESeq analysis with “fit-only” for sharing mode (FDR < 0.01). The DEGs were further filtered based on RPKM and bias values (RPKM > 1.0, bias >2). Furthermore, Kumemaru-specific DEGs were detected by comparing between cv. Kumemaru and each predominantly female-producing cultivar with edgeR (Robinson et al., 2010; McCarthy et al., 2012) using the paired-test option depending on the positions in branches (*N* = 2 × 14 for biological replicates). The DEGs were filtered according to RPKM and *p*-values (RPKM > 1.0, *p* < 0.1 for cvs. Nagara and Ogosho, and *p* < 0.05 for the rest of the 12 cultivars) (Supplementary Table 1). Putative functions of each gene were annotated with a BLASTX search of the TAIR10 database.^7^

### Construction of the Co-expression Network

The genes detected as DEGs in the 14 comparisons of predominantly female-producing cultivars and cv. Kumemaru (*N* = 20,216) were selected to construct a gene co-expression network with the WGCNA package (Langfelder and Horvath, 2008). The soft-thresholding power for a signed network was set at 5, with a scale-free model fitting index *R*^2^ > 0.6. A relatively large minimum module size (30) and a medium sensitivity (deepSplit = 2) to cluster splitting were applied. In co-expression networking, genes were represented by nodes, and the correlation values (weight) between two genes were calculated by raising the Pearson’s correlation coefficient. The heatmap for expression was designed with ggplot2 packages (Csardi and Nepusz, 2006; Wickham, 2009). The genes in the same module were first visualized with the Cytospace program (Shannon et al., 2003) and only lines with a weight greater than 0.43.

### Cataloging of cv. Kumemaru-Specific Subsequences From mRNA-Seq Reads

For the comprehensive caption of Kumemaru-specific polymorphisms in the expressed transcripts, we conducted “kmer cataloging” in the cDNA reads, according to the previous reports characterizing Y-linked polymorphisms in persimmon (Akagi et al., 2014) and kiwifruit (Akagi et al., 2018). We cataloged 35-bp subsequences triggered with an “A” nucleotide in the mRNA-Seq reads of cv. Kumemaru and of the predominantly female-producing cultivars pools using custom Python scripts.^8^ The subsequence catalogs from cv. Kumemaru and from female cultivars were compared to extract the Kumemaru-specific subsequences (“0” counts in the female cultivars pool). The Illumina reads including cv. Kumemaru-specific subsequences (k-mers) (KSK) were assembled with a CLC assembler, as described (Akagi et al., 2014). The derived contigs were filtered with the remapping of the KSK to exclude minor polymorphisms or sequencing noises (nos. of KSK < 10).

## Results

### Morphological Characterization of Male Flowers in cv. Kumemaru

**FIGURE 2:**
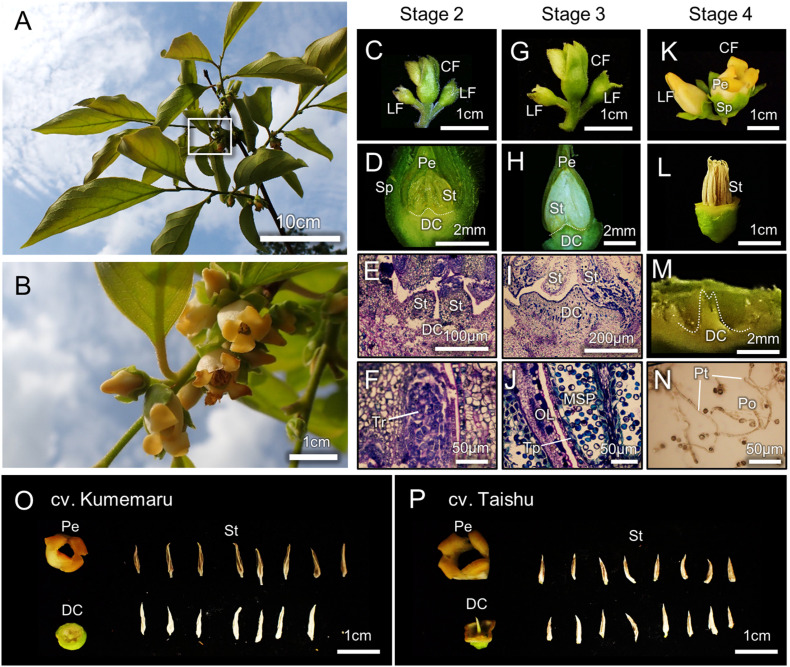
Morphological characterization of male flower development in cv. Kumemaru. **(A,B)** The appearance of flowering branches in cv. Kumemaru **(A)**, and the closing up of the white box in panel **(A)**, showing cyme-like trifurcated inflorescence **(B)**. **(C–N)** The developmental processes of male flowers in cv. Kumemaru, in the stages defined according to Yang et al. (2019). In stage 2 **(C–F)**, tetrads (Tr) were formed inside stamens (St) **(F)**. In stage 3 **(G–J)**, microspores (MSP) were developed, while tapetal layers (Tp) have not been degenerated **(J)**. In stage 4 **(K–N)**, flowers opened with mature stamens, producing fertile pollens (Po). Sp, sepal; Pe, petal; DC, deficient carpel; Pt, pollen tube. **(O,P)** Alignment of male flower components in cv. Kumemaru **(O)** and monoecious cv. Taishu **(P)**, which included four gamosepalous petals, 1 deficient carpel, and ca 16 stamens.

The architectures of flowering branches and inflorescences in cv. Kumemaru were similar to those in other monoecious cultivars, where male flowers set in medial positions of the parental branches, with cyme-like trifurcated inflorescence (Figures 2A,B). In the flower developing/maturing stages (stages 1–4) previously defined (Yang et al., 2019), developmental processes of male flowers/organs in cv. Kumemaru were consistent with other monoecious cultivars (Figures 2C–N, compared with Yang et al., 2019), producing fertile anther/pollens (Figures 2F,J,N). Flower structure/components in cv. Kumemaru were identical to male flowers in monoecious cv. Taishu (Figures 2O,P). These observations suggested that the mechanism of male flower production in cv. Kumemaru is consistent with monoecious cultivars, where gene networks orchestrated by *MeGI* play an important role (Yang et al., 2019).

### Stable Male Production System Caused by Derepression of the *OGI* Silencing

**FIGURE 3:**
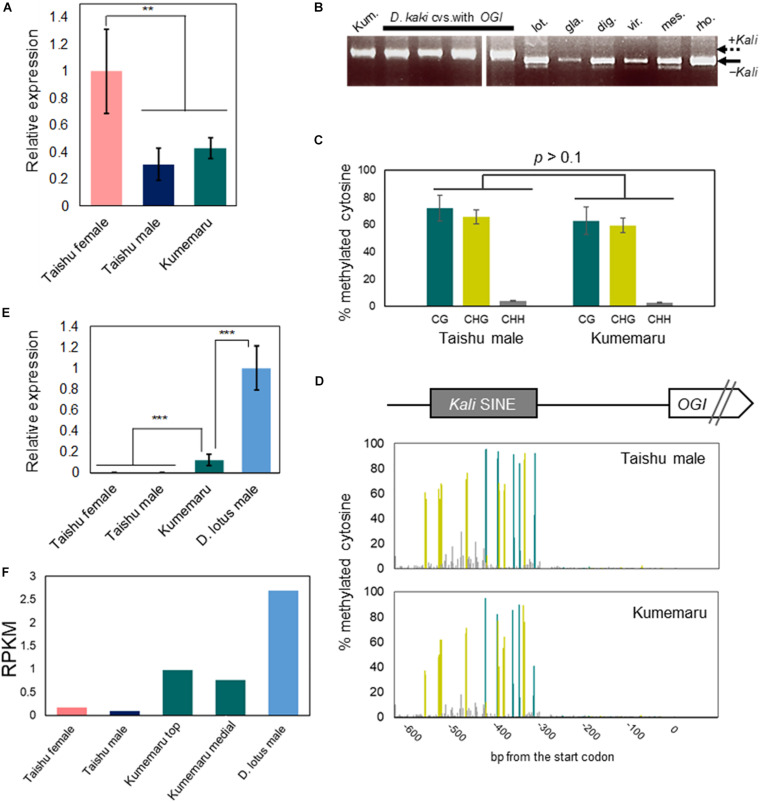
Regulation of *OGI* and *MeGI* in cv. Kumemaru. **(A)** *MeGI* expression level in flower buds in cv. Kumemaru, and monoecious cv. Taishu, from qRT-PCR analysis. cv. Kumemaru showed substantial reduction in the *MeGI* expression, similar to male flowers of cv. Taishu (**p < 0.01, *N* = 4 for biological replicates). **(B)** PCR analysis detecting the *Kali* SINE in *D. kaki*, and the relatives. cv. Kumemaru (Kum.) carries only the Kali inserted *OGI*, as well as other *D. kaki* cultivars (Akagi et al., 2016a). Other representative *Diospyros* species (lot. *D. lotus*, gla: *D. glaucifolia*, dig: *D. digyna*, vir: *D. virginiana*, mes: *D. mespiliformis*, and rho: *D. rhombifolia*) showed no *Kali*-SINE insertion in the *OGI* promoter region. **(C,D)** Cytosine methylation level **(C)** and methylation pattern at each cytosine residue **(D)** in the cv. Kumemaru and cv. Taishu *OGI* promoter region, in flower primordia. cv. Kumemaru showed similar methylation pattern with cv. Taishu (*p* > 0.1) **(E,F)** *OGI* expression level in flower buds in cvs. Kumemaru, Taishu, and diploid male *D. lotus* (cv. Kunsenshi-male), from qRT-PCR **(E)** and mRNA-Seq **(F)** analyses. *OGI* is not fundamentally expressed in monoecious cultivars including cv. Taishu (Akagi et al., 2016a), while cv. Kumemaru showed significant increase in the *OGI* expression (**E**, ***p < 0.001, *N* = 4 for biological replicates). The *OGI* expression in cv. Kumemaru was still lower than in diploid *D. lotus*, which carries no *Kali* SINE in the *OGI* promoter **(E)** (***p < 0.001, *N* = 4 for biological replicates). Bars indicate standard errors.

To find out the basic molecular mechanism of stable male production in cv. Kumemaru, we first analyzed the regulations of *OGI* and *MeGI* in cv. Kumemaru. The expression level of *MeGI* in cv. Kumemaru was substantially lower than in the predominantly female-producing cultivars, at the onset of primordia development (Figure 3A), which was consistent with male flower buds in other monoecious cultivars (Akagi et al., 2016a). For the structure of *OGI*, cv. Kumemaru has the *Kali*-SINE insertion on the *OGI* promoter like in other cultivars (Figure 3B) and simplex *OGI*. The sequences of *Kali*-SINE insertion in the promoter and transcript region showed no specific polymorphisms in comparison to other cultivars (Supplementary Figure 1; Akagi et al., 2016b). Furthermore, bisulfite PCR-Seq analysis targeting the *OGI* promoter in flower bud primordia showed that the cytosine residues in the *Kali-*SINE insertion were highly methylated in the CG and CHG sequences (62.8 and 59.4%, respectively) (Figure 3C). The methylation pattern at each cytosine residue was also consistent with male flower buds in the monoecious cultivar (*p* > 0.1; Akagi et al., 2016a) (Figure 3D). These results signified “repression” of the *OGI* expression, as observed in other cultivars (Akagi et al., 2016a). However, in cv. Kumemaru, *OGI* exhibited slight but fundamental expression in the flower bud primordia (RPKM = 0.87 and 1.18 for independent buds from different positions in mRNA-Seq analysis), which was significantly higher than in other monoecious cultivars (RPKM < 0.2 in mRNA-Seq analysis) (Figures 3E,F). The expression level of *OGI* in cv. Kumemaru was still lower than that in diploid male Caucasian persimmon (*D. lotus*) (average RPKM = 2.69), which carries no *Kali*-SINE insertion on the promoter (Figure 3F). In the late-developing stage, consistent with the diploid male persimmon, the expression levels of *OGI* in buds and other tissues were substantially decreased, suggesting that derepression of *OGI* in cv. Kumemaru is not constitutive (Supplementary Figure 2). These results suggested that the epigenetic sex regulation mechanism, in which the expression of *OGI* is suppressed under the control of the *Kali*-SINE, is shared in common among monoecious hexaploid persimmon cultivars, but *OGI* is induced with an unknown mechanism in cv. Kumemaru.

### Differentially Expressed Genes in cv. Kumemaru-Specific Manner

**FIGURE 4:**
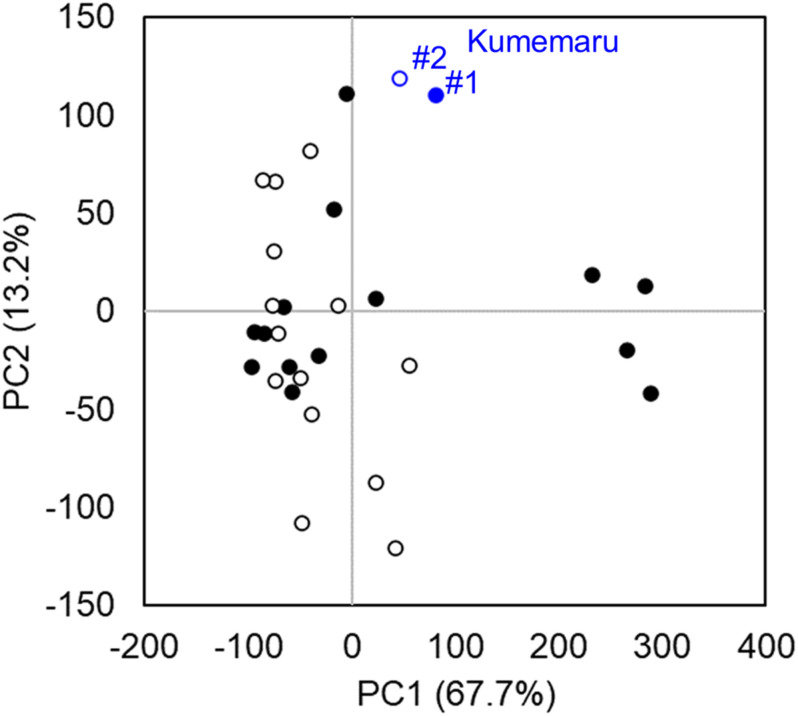
Transcriptomic profiles in comparison to cv. Kumemaru (Km) and 14 predominantly female-producing trees. Characterization of gene expression dynamics by PCA on transcriptomic data (RPKM > 1) of each cultivar. The buds were sampled from two positions, substantially affecting flower sexuality in monoecious cultivars (Akagi et al., 2016a). The two positions of buds in branches such as medial (#1, filled circle) and top (#2, open circle) based on the criteria in the previous report (Akagi et al., 2016a) were used as replicates in each cultivar. No significant difference was observed between the cv. Kumemaru and the other dominantly female-producing cultivars (*p* > 0.05).

To screen the genes associated with the mechanism for derepression of *OGI*, we compared mRNA-Seq data from the flower bud primordia in cv. Kumemaru and the 14 predominantly female-producing cultivars. Profiling with PCA using all the genes with RPKM > 1 (*N* = 32,143) indicated no specific clusters between cv. Kumemaru and the female-producing cultivars, although buds from some female-producing cultivars formed a specific cluster differentiated in the PC1 axis (Figure 4). These results indicated that cv. Kumemaru showed no dynamic expression differentiation from the female-producing cultivars.

To analyze sex-biased gene expression, we first conducted DESeq analysis between two groups, namely cv. Kumemaru vs. all 14 female-producing cultivars (*N* = 2 vs. 28 for biological replicates), defined as “comparison I.” We identified 3,441 upregulated and 2,931 downregulated DEGs (bias > 2, FDR < 0.01, RPKM > 1). These detected DEGs were annotated with gene ontology (GO) terms (FDR < 0.05). The 3,441 upregulated DEGs in cv. Kumemaru showed significantly enriched GO terms of protein phosphorylation and tyrosine kinase signaling (*p* = 1.9E-15 and 2.2E-17, respectively; Supplementary Table 2). The 2,931 downregulated DEGs in cv. Kumemaru showed enriched GO terms of post-embryonic development and response to stimulus (*p* = 1.4E-24 and 1.0E-18, respectively; Supplementary Table 3). Next, we conducted paired edgeR analysis between cv. Kumemaru and each female-producing cultivar, separately in 14 combinations (*N* = 2 × 2 for biological replicates in all 14 combinations), to detect cv. Kumemaru-specific expressions in more stringent criteria, defined as “comparison II.” We identified 21 genes that were upregulated in cv. Kumemaru in all 14 combinations and 21 genes showing downregulation (*p* < 0.1 for cvs. Nagara and Ogosho, *p* < 0.05 for the other 12 female cultivars, RPKM > 1) (Supplementary Table 4). For these DEGs, no significantly enriched GO terms were detected, presumably due to small numbers of the DEGs. Taken together, we detected the candidate genes related to the derepressed *OGI* silencing, which probably acts as an upstream of *MeGI* during early primordia differentiation.

### Core Gene Networks Correlated With the Derepressed *OGI* Expression System in cv. Kumemaru

**FIGURE 5:**
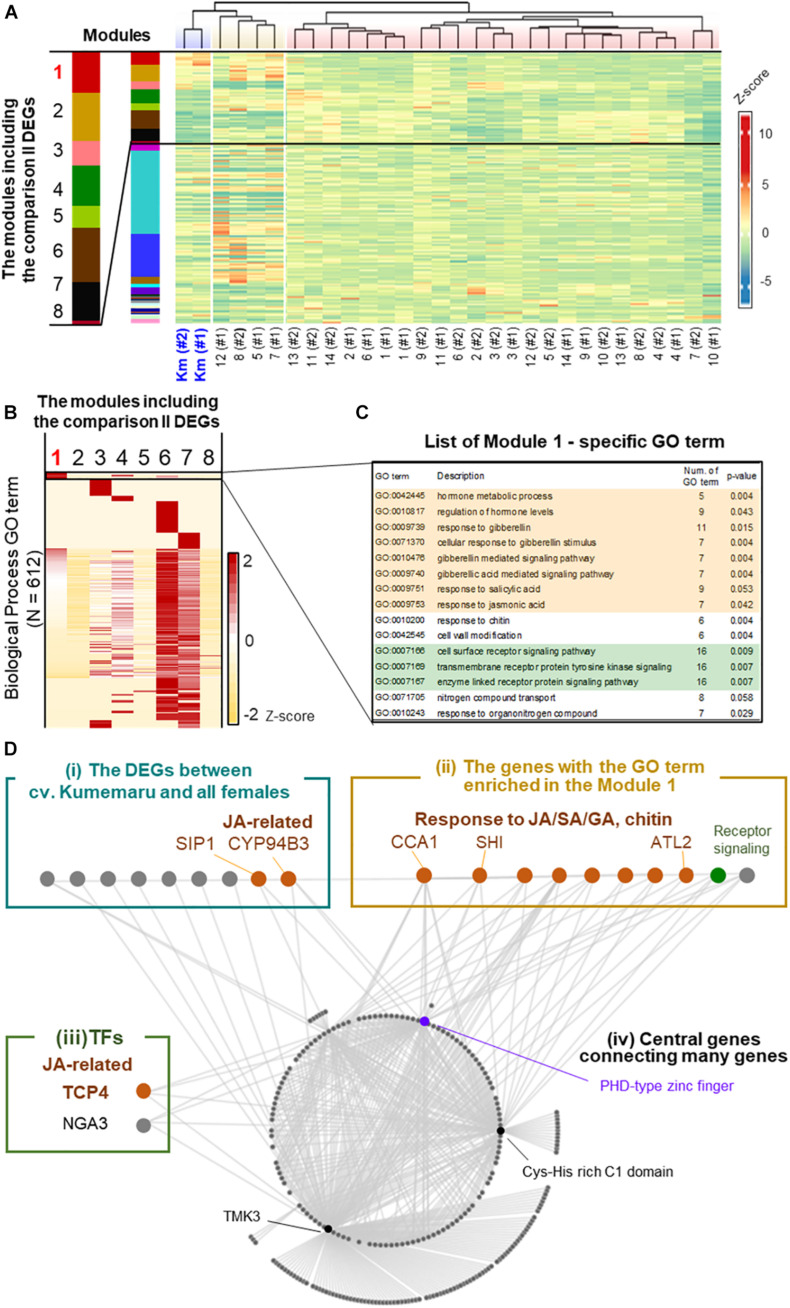
Co-expression network analysis in cv. Kumemaru and the predominantly female-producing cultivars. **(A)** Co-expression moduling of the genes based on expression patterns in cv. Kumemaru and 14 predominantly female-producing cultivars, with the WGCNA package. Modules 1–8 included the comparison II DEGs. **(B)** Heatmap for the enrichment of GO terms involving biological processes in these eight modules. The numbers of annotated GO terms were standardized by *Z*-scoring. **(C)** List of the GO terms specifically enriched in module 1 (*p* < 0.06), which included most of the cv. Kumemaru-specific DEGs. The GO terms relating to the hormonal process and receptor signaling pathway were highlighted in orange and green, respectively. **(D)** Visualization of the coexpressed gene network in module 1. As described in the main text, they were categorized into the following four groups: (i) the DEGs specific to cv. Kumemaru, (ii) the genes with the GO terms specifically enriched in module 1, as given in the panel **(C)**, (iii) transcription factors, and (iv) central genes in this network.

To understand the regulatory paths of Kumemaru-specific derepression of *OGI*, the co-expression patterns were visualized by applying a weighted correlation network analysis (WGCNA), which were first clustered into 27 modules (Figure 5A). Of them, 26 and 8 modules included the Kumemaru-specific DEGs detected from comparisons I and II, respectively (see Supplementary Table 5 for the details). Here we focused on module 1 (*N* = 993), which has the most significant enrichment of the DEGs for both comparison I (*N* = 840) and comparison II (*N* = 17) (*p* = 3.5e-519 and 2.5e-19, respectively, for one-sided Fisher’s exact test). Of the 612 biological process GO terms annotated to the genes in the 8 modules with the comparison II DEGs, 15 terms were specifically enriched in module 1 (*p* < 0.06) (Figures 5B,C). These terms included tyrosine kinase signaling pathway and response to gibberellin (GA), salicylic acid (SA), jasmonic acid (JA), and chitin (*p* < 0.06; Figures 5B,C), which overall implied association with stress signals (Xu et al., 1998; Devoto and Turner, 2004; Hu et al., 2017). These JA/GA/SA-associated genes were not included in the DEGs between male and female buds in diploid dioecious *D. lotus*, where *OGI* is genetically active in male (Akagi et al., 2014). Thus, these JA/SA/GA associated genes might contribute to the derepression of *OGI* in a cv. Kumemaru-specific manner.

To further dissect the key structure potentially derepressing *OGI* in cv. Kumemaru, we visualized the co-expression gene network in module 1 and focused on the genes highly correlated to other genes (correlation values >0.43) (Figure 5D). Here we featured the following four categories: (i) the Kumemaru-specific DEGs in comparison II, which were thought to be potential candidates to regulate Kumemaru-specific stable maleness (from 42 DEGs as described); (ii) the genes with the GO terms specifically enriched in module 1, which are mostly associated with stress signal (Supplementary Table 6) and would reflect cv. Kumemaru-specific physiological reactions; (iii) transcription factors; and (iv) central genes connecting many genes in this module. In category (i), 9 out of 17 DEGs were visualized with high correlation values with other genes (Supplementary Table 7). Among them, SIP1 mediates the abiotic stress-induced accumulation of raffinose (Egert et al., 2013), and CYP94B3 is a key enzyme in the catalytic pathway of JA biosynthesis (Heitz et al., 2011). In category (ii), focusing on JA/SA signaling, *CIRCADIAN CLOCK-ASSOCIATED* 1 (*CCA1*) and *ARABIDOPSIS TOXICOS EN LEVADURA 2* (*ATL2*) can act for the promotion of plant defense presumably via JA/SA signals (Salinas-Mondragón et al., 1999; Serrano and Guzmán, 2004; Lei et al., 2019). *ATL2* is often applied as a biomarker for JA/SA signals (Serrano and Guzmán, 2004). Focusing on GA signaling, *SHORT INTERNODES* (*SHI*) is thought to involve gibberellin response to regulate internode length and structures (Fridborg et al., 2001). The other seven genes were annotated with the GO term associated with the regulation of hormone levels, receptor signaling, and nitrogen compound transport (Supplementary Table 6). For category (iii), we found two transcription factors in module 1. TCP4 can affect JA regulation by directly controlling the expression of *LIPOXYGENASE2* (*LOX2*), which is one of the first steps of the JA biosynthesis pathway in Arabidopsis (Schommer et al., 2008). Moreover, the class II TCP family, including TCP4, was reported to regulate the expression of the other TF in category (iii), *NGATHA3* (*NGA3*) (Ballester et al., 2015). The results so far are reminiscent of the involvement of stress signals, especially with the effect of JA signals. As the core genes [or category (iv)] predominantly connecting the module 1 network (with >50 connections), the genes annotated as the PHD-type zinc finger (Lee et al., 2009), Cys-His rich C1 domain (Bhaskar et al., 2015), and TMK3 (Dai et al., 2013) were identified. Among the three genes, the PHD-zinc finger has been reported to be associated with both gene expression and repression through histone modification (Mellor, 2006), which is possibly related to *OGI* derepression.

### Identification of cv. Kumemaru-Specific Transcript Structure and Polymorphisms

To identify candidate genetic factors related to the derepression of *OGI*, kmer cataloging was conducted for the comprehensive caption of Kumemaru-specific polymorphisms. Assembly reads of Kumemaru-specific k-mers (KSK) derived 1,446 contigs (Table 1 for the representative 25 genes with >80 KSK; Supplementary Table 8 for the others). These polymorphic (or cv. Kumemaru-specific) contigs showed no overlap with the Kumemaru-specific DEGs described above. The transcript contig with the most KSK showed no homologous sequences in the persimmon genome (see text footnote 5; Akagi et al., 2020), while it showed undefined numerous virus-like sequences similar to marafiviruses. For other transcript contigs with fundamental numbers of the KSK, we could identify a structural variation of 38-bp insertion in RING-type E3 ubiquitin ligase. The other candidate contigs with the KSK showed no clear disruptions directly related to their protein functions.

**TABLE 1:** List of the genes including cv. Kumemaru-specific k-mers (KSK) with >80 KSK.

**Contigs**	**KSK counts**	**Annotation**		**Highest hit in blastx with TAIR (or with nr database for no hits in TAIR)**	
		**By persimmon DB**	**By TAIR**		**e-value**
Contig_19	1428	No hit	No hit	Blackberry virus S (*Marafivirus)*	0
Contig_492	140	No hit	No hit	Persimmon virus A (*Cytorhabdovirus)*	2E-158
Contig_905	133	Dlo_pri 0061 F.1_g 04290.1	AT4G19670.7	RING/U-box superfamily protein	0
Contig_171	128	Dlo_pri0084F.1_g01740.1	AT5G49910.1	Chloroplast heat shock protein 70-2	0
Contig_103	122	Dlo_pri0026F.1_g01940.1	AT5G45650.2	Subtilase family protein	0
Contig_841	119	Dlo_pri0002F. 1_g02880.1	AT1G79570.2	Kinase with octicosapeptide/Phox/Bem1p domain-containing	0
Contig_634	117	Dlo_pri0213F.1_g 00220.1	AT1G10430.2	Protein phosphatase 2A-2	0
Contig_221	111	Dlo_pri0044F1_g 02210.1	AT3G09630.1	Ribosomal protein L4/L1 family	0
Contig_739	109	Dlo_pri0176F1_g01650.1	AT5G62770.1	Membrane-associated kinase regulator%2C putative	3E-27
Contig_495	106	No hit	No hit	Nectarine marafivirus M (*Marafivirus)*	8E-42
Contig_275	104	Dlo_pri0004F. 1_g05490.1	AT3G16910.1	Acyl-activating enzyme 7	0
Contig_131	98	Dlo_pri0002F1_g 00210.1	No hit	No hit	
Contig_314	97	Dlo_pri0453F. 1_g00560.1	AT1G28520.5	Vascular plant one zinc finger protein	0
Contig_72	96	Dlo_pri0050F. 1_g02250.1	AT5G50360.1	Von Willebrand factor A domain protein	2E-84
Contig_114	93	Dlo_pri0072F1_g 00140.1	AT2G44730.1	Alcohol dehydrogenase transcription factor Myb/SANT-like family	2E-18
Contig_1165	93	Dlo_pri0890F-1.1_g00030.1	AT1G33970.3	P-loop containing nucleoside triphosphate hydrolases superfamily	e-119
Contig_576	93	Dlo_pri0015F1_g 00060.1	AT4G31880.2	Transcriptional regulator	e-147
Contig_292	90	Dlo_pri0225F1_g01580.1	AT2G44260.1	DUF946 family protein (DUF946)	0
Contig_319	90	Dlo_pri0138F1_g01600.1	AT4G24220.1	NAD(P)-binding Rossmann-fold superfamily protein	0
Contig_1778	89	Dlo_pri0606F. 1_g00080.1	AT4G16143.2	Importin alpha isoform 2	0
Contig_230	89	Dlo_pri0133F1_g01490.1	AT1G67 430.1	Ribosomal protein L22p/L17e family protein	e-116
Contig_324	83	Dlo_pri0198F1_g 00560.1	AT4G02590.2	Basic helix-loop-helix (bHLH) DNA-binding superfamily protein	2E-79
Contig_518	82	Dlo_pri0037F. 1_g04600.1	AT1G 12570.1	Glucose-methanol-choline (GMC) oxidoreductase family protein	0
Contig_594	82	Dlo_pri0439F. 1_g00380.1	AT5G43810.4	Stabilizer of iron transporter SufD/Polynucleotidyl transferase	0
Contig_105	81	Dlo_pri0337F. 1_g00950.1	AT3G 14240.1	Subtilase family protein	0

## Discussion

Our result of DEGs and co-expression network, focusing on the cv. Kumemaru-specific upregulated module, suggested the involvement of stress, particularly the JA-related pathway, for the activation of *OGI* expression in cv. Kumemaru. In addition to the genes involved in regulations of stress-induced plant hormones, other keywords (or GO terms in module 1) including chitin response and/or tyrosine kinase signaling (see Figures 5B,C) would imply the involvement of plant defenses and immunity. Tyrosine kinase has been reported to be responsible for biotic or abiotic stresses (Miyamoto et al., 2019) and is also known as a regulator of gibberellin responses (Fu et al., 2002), abscisic acid (ABA) signaling (Ghelis et al., 2008), cold stress (Sangwan et al., 2001), and sugar responses (Ritsema et al., 2009). Furthermore, one of the core genes in module 1, *TMK3*, as an Auxin-Binding Protein (ABP) family gene (Xu et al., 2014), is a member of the transmembrane kinase *TMK* subfamily, which is reported to be inducible under the stress or JA signals in *Nicotiana* plants (Cho and Pai, 2000). These implications might be consistent with empirical observations of flower sex bias in a tree in monoecious persimmon cultivars. Monoecious trees are supposed to produce only female flowers in vigor branches and/or at young age, while older or stressed branches tend to produce a high frequency of male flowers (Hasegawa, 1995; Hasegawa et al., 2004), although they might depend on cultivars/accessions. Furthermore, the ratio of male/female flowers in trees depends on their parental branch characters, such as sexuality, length, and bud positions (Akagi et al., 2016a). These reports suggest that not only genetic factors but also internal and external conditions of cv. Kumemaru may be involved in stresses, including specific viral infection as showed in Table 1, and may be the causal factor(s) to express derepressed male production. The viral-like sequence detected in this study was not matched to any other known viruses but most likely to be classified into the genus *Marafivirus*. Although the pathogenicity of this virus is little known, viral proteins encoded by another plant virus are thought to often inhibit JA signaling in Arabidopsis thaliana plants (Lozano-Duran et al., 2011) or to interact with plant histone deacetylase (Wang et al., 2018). Thus, it may be possible that virus infection can induce specific stress signaling to activate *OGI*, although we need further investigation to validate that in the future.

**FIGURE 6:**
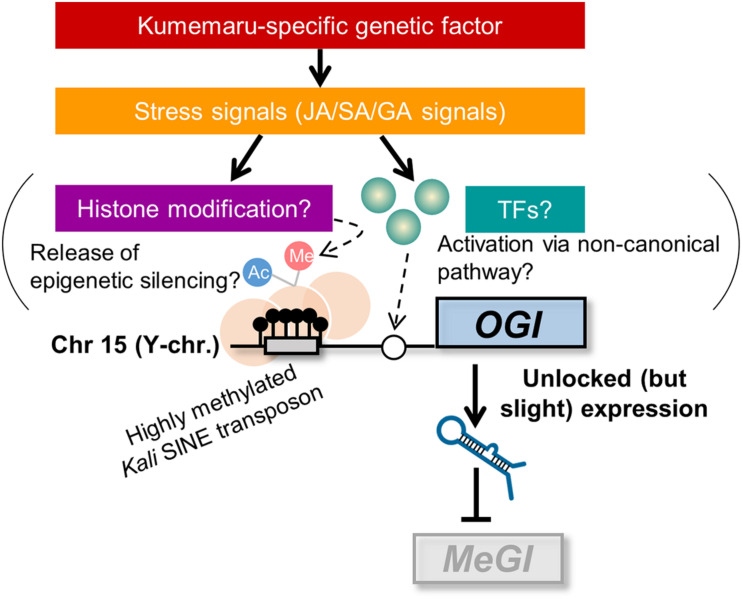
Model for derepressed O*GI* expression in cv. Kumemaru. Inactivation of *OGI* due to the *Kali*-SINE insertion and the resultant DNA methylation can be activated by two potential pathways: (i) release from the epigenetic silencing, which might result from histone modification, and (ii) an undefined “non-canonical” pathway such as activation via cv. Kumemaru-specific transcription factors. Those are likely induced by stress signals, especially JA/SA/GA signals, under the control of hypothetical Kumemaru-specific genetic (or possibly environmental) factors.

The direct effectors of derepressed *OGI* still remain to be solved. We can propose mainly two potential pathways to activate *OGI*: (i) release from the repression by the *Kali*-SINE insertion on the promoter region and (ii) an undefined “non-canonical” pathway that other monoecious cultivars do not have and cv. Kumemaru specifically established (Figure 6). For possibility (i), the *Kali*-SINE was highly methylated in cv. Kumemaru, as well as other cultivars (Figure 3D; Akagi et al., 2016a), suggesting that epigenetic conditions other than DNA methylation, such as histone methylation/acetylation, might affect the *OGI* expression. These histone modifications are frequently associated with the condition of DNA methylation on transposons (Qian et al., 2012; Du et al., 2015; Zhang et al., 2018). Although we have not assessed the histone methylation/acetylation in the *OGI* promoter, a comparison of them among a wide variety of cultivars may unveil the importance of histone modification. For possibility (ii), transcription factors that were highly expressed in cv. Kumemaru and related to JA signals such as TCP4 and NGA3 (Figure 5D) might be candidate *trans*-factors to regulate the non-canonical pathway to activate *OGI*, although k-mer cataloging has not found cv. Kumemaru-specific polymorphisms on them. On the other hand, cv. Kumemaru-specific mutations in *cis*-factors (or mutations in the promoter sequences) would be unlikely to be the causal factor to establish a derepressed *OGI* expression system. Mutations in *cis*-factors of *OGI* would affect the regulatory paths only under the control of *OGI* (or targeted *MeGI*), while the JA/SA/GA-related genes detected as DEGs specific to cv. Kumemaru are thought not to be the downstream pathways of the *OGI* gene (Akagi et al., 2014; Yang et al., 2019). As given in the model (Figure 6), future assessment about the factors potentially connecting stress signals and the *OGI* release would have importance to reveal the mechanisms not only of the cv. Kumemaru-specific derepressed male producing system but also for the evolution of monoecious system in a wide variety of hexaploid persimmon cultivars.

## Data Availability Statement

All Illumina sequencing data have been deposited in the DDBJ database: Short Read Archives (SRA) database (BioProject ID PRJDB9564, Run ID DRR233540-DRR233569).

## Author Contributions

TA designed the study. KM, HY, and TA conducted the experiments. KM, NF, and TA performed the data analyses and wrote the manuscript. KU, YK, and RT contributed to plant resources and facilities. All authors have read and approved the final manuscript.

## Conflict of Interest

The authors declare that the research was conducted in the absence of any commercial or financial relationships that could be construed as a potential conflict of interest.

## Footnotes

1 https://www.gene.affrc.go.jp/databases_en.php?section=plant

2 https://www.gene.affrc.go.jp/databases-plant_search_detail_en.php?jp=117219

3 http://qb3.berkeley.edu/gsl/

4 https://github.com/Comai-Lab/allprep/blob/master/allprep-13.py

5 http://persimmon.kazusa.or.jp/index.html

6 https://github.com/Ih3/bwa

7 https://www.arabidopsis.org/index.jsp

8 https://github.com/Comai-Lab/kmer-extract-by-trigger-site
