# Supplementary material for: Molecular Mechanism Underlying Derepressed Male Production in Hexaploid Persimmon
Source: Front Plant Sci. 2020 Dec 22;11:567249. doi: 10.3389/fpls.2020.567249 (PMC7783364; doi:10.3389/fpls.2020.567249)
Supplement: Supplementary file 3 [file Data_Sheet_3.DOCX]

Figure S1

**Figure S1. Comparison of the Kali-SINE insertion in *OGI* promoter**

Nucleotide sequence alignments of *Kali*-SINE with other *D.kaki* cultivars. There are 3 single nucleotide polymorphisms (SNP) in the *Kali*-SINE insertion. The *Kali*-SINE sequence of cv. Kumemaru was similar to other cultivars.

Figure S2

**Figure S2. *OGI* expression level in flower buds during two developing stage**

*OGI* expression level in flower buds at different two stages (developing and late-developing stage) in cvs. Kumemaru, Taishu, and diploid male *D. lotus* (cv. Kunsenshi-male) from qRT-PCR analyses. The expression levels of *OGI* were decreased in late-developing stage, which was consistent with that in diploid male persimmon.

Table S1 List of plant material and the number of the DEGs

Table S2 List of enriched GO terms in the up-regulated DEGs

Table S3 List of enriched GO terms in the down-regulated DEGs

Table S4 List of the comparison II Kumemaru-specific DEGs

See Excel data (Table S4.xlsx)

Table S5 The number of the Kumemaru-specific DEGs detected from the comparison I and II in 27 modules

Table S6 List of the genes with the GO terms specifically enriched in module 1

Table S7 List of the comparison II Kumemaru-specific DEGs in module 1

Table S8 List of the genes including cv. Kumemaru specific k-mers

See Excel data (Table S8.xlsx)
